# Comparison of neural signal sources for discriminating “crave” and “don’t crave” task conditions: Implications for fMRI neurofeedback

**DOI:** 10.1162/IMAG.a.1277

**Published:** 2026-06-18

**Authors:** Dong-Youl Kim, Jonathan Lisinski, Brooks Casas, Stephen LaConte, Pearl H. Chiu

**Affiliations:** Fralin Biomedical Research Institute at VTC, Virginia Tech, Roanoke, VA, United States; Department of Psychology, Virginia Tech, Blacksburg, VA, United States; Department of Biomedical Engineering and Mechanics, Virginia Tech, Blacksburg, VA, United States

**Keywords:** smoking craving, neurofeedback, real-time fMRI, support vector machine, classification, signal sources

## Abstract

Numerous prior studies have shown that neural features can be voluntarily modulated through real-time functional magnetic resonance imaging (rtfMRI)-based neurofeedback (NF), and that such modulation can influence cognitive and affective states, as well as improve psychiatric and neurological symptoms. To generate neurofeedback, these studies have leveraged information from a variety of signal sources, including neural activity within regions-of-interest (ROIs) and machine learning (ML)-based multivariate networks; however, little work has evaluated the relative performance of differing sources. In this study, based on the premise that improved differentiation between task conditions ought to improve neurofeedback, and to provide a foundation for future neurofeedback studies, we evaluated the performance of four different neural signal sources for discriminating among “crave” and “don’t crave” task conditions using our previously published data in which participants regulated cigarette craving across rtfMRI-NF runs. Thirty-one smokers performed three runs including an initial run for training a baseline ML model and subsequent run for testing the baseline model, as well as an updated model from the second run being tested during the third run. The ML NF signal reflected the distance to the hyperplane of a support vector machine classifying “crave” and “don’t crave” conditions. To compare the performance of different neural signal sources for discriminating task conditions over repeated runs, we evaluated within-ROI neural activity, between-ROIs functional connectivity, activity-based classification accuracy, and connectivity-based classification accuracy for differentiating “crave” and “don’t crave” task conditions over runs. ML-based sources showed better performance for discriminating conditions than non-ML-based sources, with connectivity-based ML sources exhibiting the best performance among signal sources. Taken together, we demonstrate that multivariate machine learning-based analyses enhance discriminability of neural measures associated with smoking cravings across repeated neurofeedback training and suggest the potential for seed-based functional connectivity to further enhance discriminability.

## 1 Introduction

Many studies have provided evidence for the feasibility of voluntary modulation of functional processes in the human brain using real-time functional magnetic resonance imaging (rtfMRI)-based neurofeedback (NF) (DeCharms et al., 2005; Fede et al., 2023; Haugg et al., 2020, 2021; Kim et al., 2015; LaConte, 2011; Ros et al., 2020; Ruiz et al., 2014; Sitaram et al., 2017; Sulzer et al., 2013; Weiskopf, 2012; Young et al., 2017).

The last decade has seen growing interest in the potential application of rtfMRI-NF as a non-invasive neuromodulatory technique for the treatment of a variety of neurological and psychiatric conditions (DeCharms et al., 2005; Emmert et al., 2017; Fede et al., 2023; Morgenroth et al., 2020; Papoutsi et al., 2020; Weaver et al., 2020; Young et al., 2017). In the area of smoking cessation interventions, rtfMRI-NF has shown promising results in regulating smoking cravings among tobacco cigarette smokers (Canterberry et al., 2013; Hartwell et al., 2016; Karch et al., 2019; Kim et al., 2015). In earlier studies (Canterberry et al., 2013; Hartwell et al., 2016), the NF signal was based on hemodynamic activity in regions-of-interest (ROIs) including the medial frontal cortex, an area implicated in cigarette cravings, evoked by visual cues associated with smoking. More recently, Karch and colleagues investigated the efficacy of modulating neural activity within ROIs including the anterior cingulate cortex (ACC), dorsolateral prefrontal cortex (DLPFC), and insula, for reducing smoking cravings using traditional activity-based rtfMRI-NF approaches (Karch et al., 2019).

Accruing evidence has implicated a network of brain regions in smoking cravings including the ACC (Azizian et al., 2010; Brody et al., 2007; Due et al., 2002; Ernst et al., 2001; Hartwell et al., 2011; Kober et al., 2010; Smolka et al., 2006), medial prefrontal cortex (mPFC) (Brody et al., 2007; Hartwell et al., 2011; Stein et al., 1998; Sutherland et al., 2012), medial orbitofrontal cortex (mOFC) (Due et al., 2002; Hartwell et al., 2011; Lee et al., 2012; Smolka et al., 2006), posterior cingulate cortex (PCC) (Brody et al., 2007; Hartwell et al., 2011; Lee et al., 2012; Stein et al., 1998), precuneus (Brody et al., 2007; Hartwell et al., 2011; Lee et al., 2012), and insula (Brody et al., 2007; Lee et al., 2012; Naqvi et al., 2007; Stein et al., 1998; Sutherland et al., 2012). By targeting individual ROIs, standard rtfMRI-NF methods have enabled participants to successfully modulate neural activity evoked by smoking-related visual cues (Canterberry et al., 2013; Hartwell et al., 2016; Karch et al., 2019). However, the efficacy of this approach may be limited by its focus on activity in individual regions, rather than making use of the multivariate patterns of activity across multiple regions implicated in craving-related cognitions.

Current rtfMRI-NF studies have also demonstrated that modulation within targeted regions-of-interest (ROIs) similarly results in modulation of neural connectivity between the ROIs or across multiple brain regions (Luo et al., 2023; Taebi et al., 2024; Taschereau-Dumouchel et al., 2022; Tsuchiyagaito et al., 2021; Zotev et al., 2024). As an example, Zotev and colleagues conducted an rtfMRI-NF study to regulate neural activity levels within the dorsolateral prefrontal cortex and reported that the functionally connected areas were modulated by rtfMRI-NF training (Zotev et al., 2024).

More recently, machine learning approaches have been used on multiple brain regions and across the whole brain volume to generate neurofeedback signals (Collin et al., 2022; Fede et al., 2023; LaConte, 2011; Lorenzetti et al., 2018; McDonald et al., 2017; Shibata et al., 2011; Weaver et al., 2020). Fede and colleagues adopted a support vector machine (SVM) approach to generate neurofeedback signals, finding better modulation performance among alcohol-dependent individuals when continuous feedback rather than intermittent feedback was used (Fede et al., 2023).

A separate line of studies has also reported that functional connectivity patterns both overlap with task-driven activity patterns (Salvo et al., 2021) and can be used to predict neural activity and related behavioral traits (Gal et al., 2022). In addition, functional connectivity-informed models predict changes of neural activity induced by motor, language, and working memory tasks (Cole et al., 2021). Given recent evidence suggesting that functional connectivity components can predict patterns of neural activity, we posited that these features could have beneficial potential as signal sources for efficacious delivery of rtfMRI-NF.

While recent rtfMRI-NF research has applied multiple techniques to provide feedback derived from various neural signal sources, including neural activity, connectivity, and machine learning-derived measures of activity and connectivity, no study to our knowledge has compared the relative performance of these signal sources for providing fMRI-based neurofeedback. Most prior studies have used a single signal source within a given neurofeedback experiment, rendering it difficult to assess whether differences in outcomes between studies reflect differences in the neural sources used to provide the neurofeedback or other differences in study design and sample characteristics. Thus, by directly comparing multiple signal sources of neurofeedback within the same dataset, our approach minimizes these confounds and allows for a clearer assessment of the relative strengths and limitations of different neural sources for neurofeedback.

We previously reported the performance of rtfMRI-NF on group- and individual-level classification of craving-related neural responses among cigarette smokers (Kim et al., 2024). Here, we leverage this same dataset to evaluate the performance of different neural signal sources for differentiating between “crave” and “don’t crave” task-related neural responses to inform optimal neural signal sources for future rtfMRI neurofeedback studies, based on the premise that better discriminability between relevant task conditions will provide a more useful neurofeedback signal. Therefore, this study provides insights that go beyond demonstrations of feasibility, offering a systematic comparison that can guide choices of neural signal sources for future rtfMRI-NF research. We further evaluate changes in neural features between pairs of repeated runs and across repeated runs depending on the neural signal source. We present this work with the caveat that these are off-line analyses of data derived from a single source neurofeedback study; the limitations of this approach and opportunities for future improvement are detailed in the discussion.

## 2 Methods and Materials

### 2.1 Participants

The study protocol was approved by the institutional review board (IRB) at Virginia Tech, and the rtfMRI-NF task design and methods were previously reported in Kim et al. (2024). The present study re-analyzes those data to assess the differentiability of “crave” vs “don’t crave” task conditions based on neural signal source. All subjects provided written consent before the experiment and were compensated based on participation duration. Eighty-seven adults (43 male, age = 32.4 ± 11.5 years) were screened through telephone interviews.

Participants were required to be between the ages 18 and 55 years, right-handed, be fluent in English, smoke at least 5 cigarettes per day, have been smoking cigarettes for at least the past year, be willing to abstain from cigarette use for at least 12 hours prior to imaging, not be currently trying to quit smoking, and be able to see the computer display clearly with MR-compatible eyeglasses. Exclusion criteria included left-handedness, claustrophobia, current diagnosis of an Axis I or II disorder based on the Diagnostic and Statistical Manual of Mental Disorders (DSM)-IV (Ap, 1994) exclusive of nicotine dependence, current pregnancy, contraindications to MRI (e.g., pacemaker, aneurysm clips, neurostimulators, cochlear implants, metal in eyes, steel worker, or other implants), active neurologic disorder, history of alcohol or drug dependence (excluding nicotine), and history of head injuries resulting in loss of consciousness more than 10 minutes.

Fifty-eight participants were eligible after telephone screening; nine of these participants withdrew prior to scanning. Data from scanned participants were excluded from the present analysis due to (a) failure to meet abstinence criteria at MRI day (n = 12) or (b) excessive head movement (absolute translation parameter in any direction > 2 mm; n = 6). Demographic and behavioral data of included participants (n = 31) are summarized in Table 1.

**Table 1.** Demographic and smoking-related characteristics of the sample.

	n = 31
Age (years)	32.97 ± 11.54
Gender (male), N (%)	15 (48.39%)
Cigarettes/day	15.03 ± 7.84
Cigarettes used yesterday	13.65 ± 6.87
FTND	4.61 ± 2.28
SJWS total score	100.87 ± 24.08
SJWS smoking items	41.74 ± 6.87
CO level in the lungs (ppm)	14.45 ± 8.05*
CO level in the blood (%Hb)	2.94 ± 1.29*

The data are presented as the mean values ± standard deviation.

* *n* = 29 due to missing data from two subjects.

FTND, Fagerström Test of Nicotine Dependence (Heatherton et al., 1991); SJWS, Shiffman–Jarvik Withdrawal Scale (Shiffman & Jarvik, 1976); breath CO, carbon monoxide.

### 2.2 Experimental setup

**Fig. 1.**
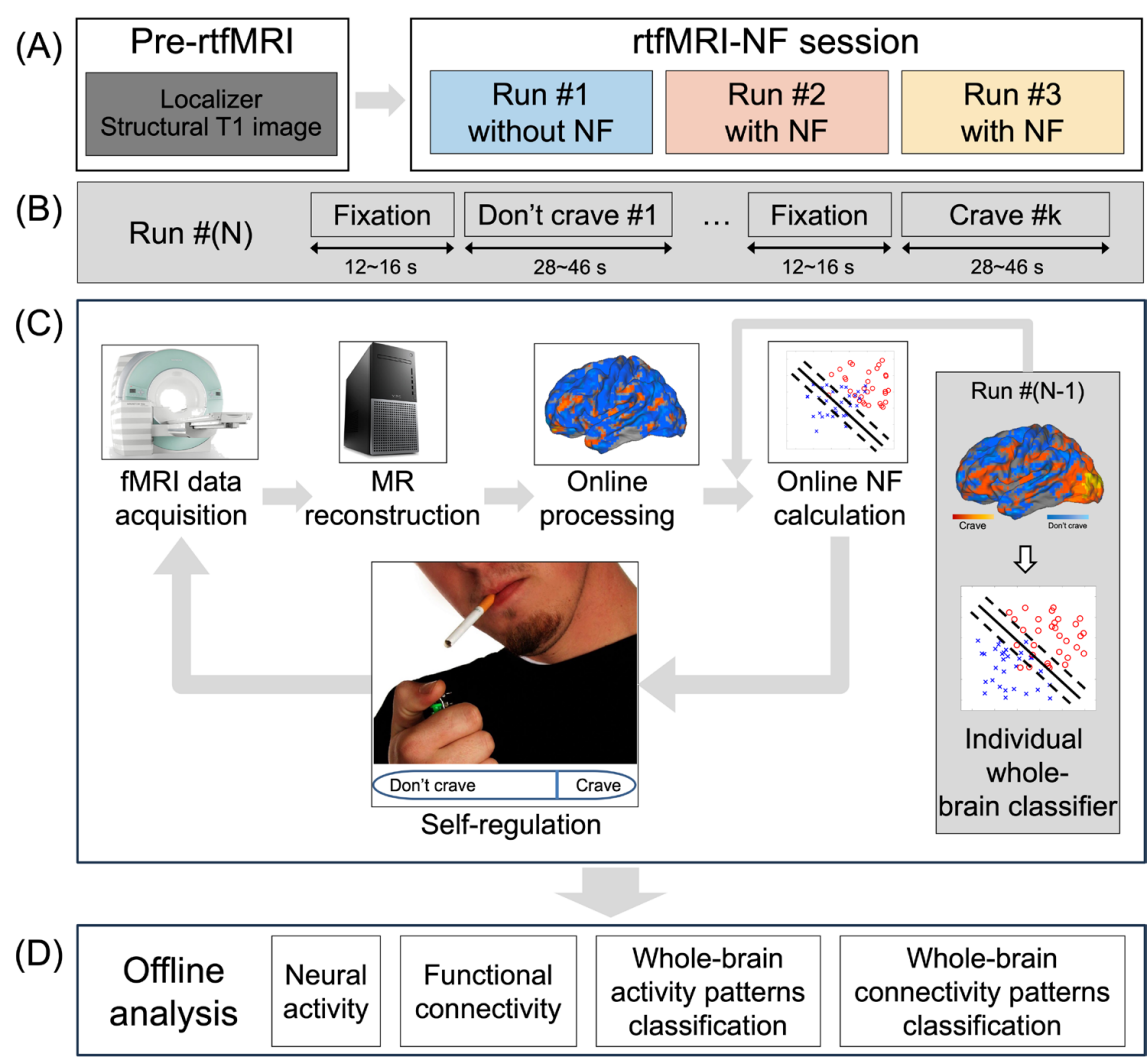
Overall experimental procedure for control of smoking craving brain patterns. (A) Procedure of MRI session during a visit, (B) rtfMRI run including *k* blocks for each of “crave” and “don’t crave” conditions, (C) information on the NF phase of each rtfMRI-NF run and how to apply the classifier from the prior run to the following run, (D) all acquired data via rtfMRI-NF experiment further used in offline analysis. rtfMRI, real-time fMRI; NF, neurofeedback.

Figure 1 illustrates the overall experimental procedure. Three rtfMRI-NF runs followed a localizer and structural run and were performed while participants viewed a series of smoking-related images (Gilbert & Rabinovich, 1999) and received ongoing instructions, based on task block, to either “crave” or “don’t crave”. The first run (684 sec) included counterbalanced “crave” and “don’t crave” blocks (n = 6 each) interleaved by fixation blocks (n = 13). During the fixation blocks, participants were instructed to “keep fixating on the crosshair, remain at rest with your eyes open, and not think of any mental strategies”. The second and third runs (768 sec each) with neurofeedback consist of 7 blocks for each “crave” and “don’t crave” condition and 15 fixation blocks. Participants could freely choose their own strategies to regulate their craving with example strategies such as “how a cigarette looks, smells, feels, and how it would taste right now” for the “crave” condition and “the bad tastes or smells or feeling unhealthy” for the “don’t crave” condition. A slider bar reflecting the neurofeedback was updated every 2 sec (i.e., at each TR volume) and presented to the participants, positioned under the images.

### 2.3 Imaging parameters

The blood-oxygen-level-dependent (BOLD) fMRI data were acquired with a standard gradient-echo echo-planar imaging (EPI) pulse sequence using a 3-T Siemens Tim-Trio scanner with a 12-channel head coil (Erlangen, Germany): time of repetition (TR) = 2000 ms, time of echo (TE) = 30 ms, field of view (FoV) = 220 × 220 mm^2^, matrix size = 64 × 64, voxel size = 3.4 × 3.4 × 4.0 mm^3^, flip angle (FA) = 90°, 33 interleaved slices. The structural T1-weighted image was acquired with the parameters: GeneRalized Autocalibrating Partial Parallel Acquisition (GRAPPA; TR = 2600 ms, TE = 3.02 ms, FoV = 256 × 256 mm^2^, voxel size = 1 × 1 × 1 mm^3^, FA = 8°, 192 sagittal slices with no gap).

### 2.4 Real-time fMRI (rtfMRI)-neurofeedback (NF) system settings

The system for rtfMRI-NF was implemented based on previous studies (LaConte, 2011; LaConte et al., 2007). Three computers for the scanner console, stimulus presentation, and real-time monitoring, respectively, were used for the system. The stimulus presentation computer was connected to the console computer of the MRI scanner, allowing raw EPI volumes reconstructed on the console computer to be available on the stimulus computer in real time. The reconstructed image data on the computer sent image data via TCP/IP to an additional computer with AFNI for real-time monitoring of motion parameters and neural patterns by the experimenter. The task paradigm was presented to participants using Vision Egg (Straw, 2008) and included a slider-bar interface reflecting the neurofeedback.

### 2.5 Generation of neurofeedback signal

For generating neurofeedback in our prior neurofeedback work (Kim et al., 2024), the localizer run (8 sec) was used to define the brain mask generated by AFNI’s 3dAutomask. To estimate the neural signals for neurofeedback, the whole-brain BOLD signals in the first run were preprocessed with slice timing correction and motion correction using AFNI. The preprocessed BOLD signals excluding the first two volumes of all blocks were defined as the input data to classify the “crave” and “don’t crave” conditions using AFNI’s 3dsvm (3d-SVM with a linear kernel; fixed parameter of SVM, C = 200). The preprocessed BOLD signals in the following run were used to distinguish the “crave” and “don’t crave” conditions in a volume-wise manner. In addition, a classifier model was updated using preprocessed BOLD signals in the second run and the preprocessed BOLD signals in the third run were used to classify the two conditions using the model from the second run. A classifier distance from the hyperplane was estimated at each TR, and positive and negative distances were used to provide the neurofeedback as a slider bar moving toward “crave” or “don’t crave” anchors at each TR, respectively. A linear trend of the distance was removed to correct for temporal drifts and mean offset. In addition, the absolute value of distance was transformed to four discrete bins (|d| < 0.2, 0.4, 0.6, or 0.8, and |d| ≥ 0.8) to avoid excessively small movements. The acquired EPI volumes from all rtfMRI-NF runs were further processed in offline analysis.

### 2.6 Offline analysis: Preprocessing of rtfMRI-NF data

As noted above, the primary goal of the present study was to leverage our prior real-time fMRI dataset in smokers to evaluate the performance of different neural signal sources for differentiating between “crave” and “don’t crave” related neural responses to inform optimal neural signal sources for future rtfMRI neurofeedback studies, based on the premise that better discriminability between relevant task conditions will provide a more useful neurofeedback signal. We further evaluate changes in these neural features between pairs of repeated runs and across repeated runs depending on the neural signal source. The steps of the offline analyses and extraction of neural data from the different sources are detailed here.

For the offline analyses for each neural source, the EPI volumes from each rtfMRI-NF run were preprocessed with head motion correction, spatial normalization to the Montreal Neurological Institute (MNI) space (isotropic 3 mm voxel size), and spatial smoothing using an 8 mm isotropic full-width at half-maximum Gaussian kernel using SPM12. In addition, temporal preprocessing steps were applied including temporal smoothing across three volume periods (3 s) followed by temporal detrending to significantly reduce low-frequency linear drift noise. The first block from the second and third runs was not further analyzed to match the number of blocks across the three runs.

### 2.7 Offline analysis: Identification of ROIs

**Fig. 2.**
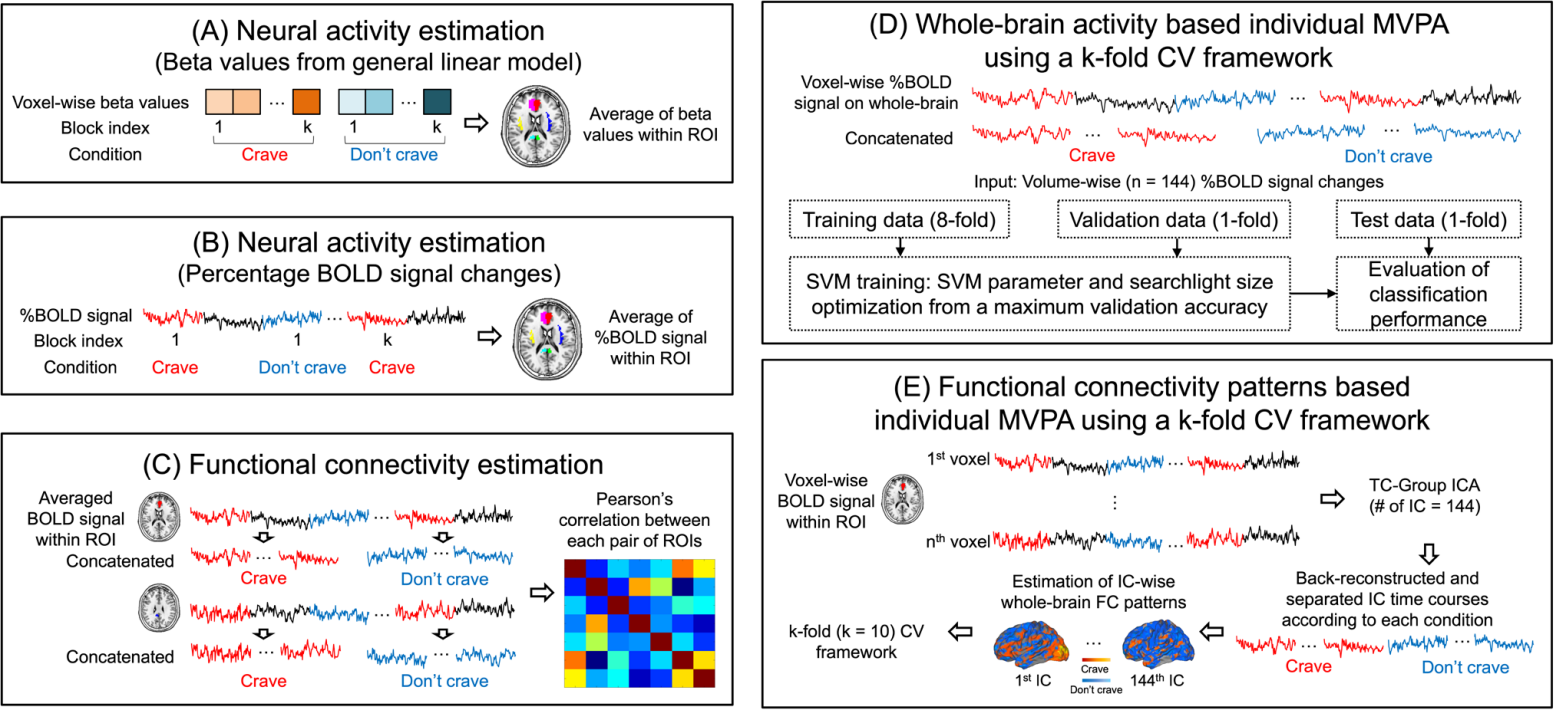
Schematic diagram for approaches to measure each signal source. (A) Neural activity estimation based on beta values from the contrast of “crave” and “don’t crave” conditions using general linear model, (B) neural activity estimation based on difference of percentage BOLD signal changes between “crave” and “don’t crave” conditions, (C) functional connectivity estimation using difference of z-scored Pearson’s correlation coefficients between “crave” and “don’t crave” conditions, (D) whole-brain activity based individual MVPA using *k*-fold CV framework to classify “crave” and “don’t crave” conditions, (E) functional connectivity patterns-based individual MVPA using *k*-fold CV framework to classify “crave” and “don’t crave” conditions. ROI, regions-of-interest; BOLD, blood-oxygen-level-dependent; MVPA, multi-voxel pattern analysis; CV, cross-validation; SVM, support vector machine; TC, temporally concatenated; ICA, independent component analysis; IC, independent component; FC, functional connectivity.

Based on previous studies to investigate neural circuits of smoking craving, multiple ROIs were defined to examine differences between “crave” and “don’t crave” conditions among four signal sources: (i) neural activity based on beta value or percent BOLD signal, (ii) functional connectivity, (iii) activity-based classification, and (iv) connectivity-based classification. Specifically, the ACC (Azizian et al., 2010; Brody et al., 2007; Due et al., 2002; Ernst et al., 2001; Hartwell et al., 2011; Kober et al., 2010; Smolka et al., 2006), medial superior frontal gyrus (mSFG) (Brody et al., 2007; Hartwell et al., 2011; Stein et al., 1998; Sutherland et al., 2012), mOFC (Due et al., 2002; Hartwell et al., 2011; Lee et al., 2012; Smolka et al., 2006), PCC (Brody et al., 2007; Hartwell et al., 2011; Lee et al., 2012; Stein et al., 1998), precuneus (Brody et al., 2007; Hartwell et al., 2011; Lee et al., 2012), and insula (Brody et al., 2007; Lee et al., 2012; Naqvi et al., 2007; Stein et al., 1998; Sutherland et al., 2012) were utilized to compare performance across signal sources. Figure 2 shows the methodology for measuring each neural signal source, with further details are described in the following paragraphs.

### 2.8 Offline analysis: Measures of neural activity

The level of neural activity was measured based on beta values and percentage BOLD signal changes (PSC) as shown in Figure 2A-B. Beta values were calculated using a general linear model (GLM) with a hemodynamic response function (HRF). The onset and duration of each condition (i.e., “crave” and “don’t crave”) were defined for each block, run, and participant using SPM12. For each participant, neural activity level was estimated from the beta values across voxels of each ROI based on the contrast of “crave” and “don’t crave” conditions. PSC was estimated using the BOLD time series of each voxel by adjusting a baseline intensity, which was defined as the average below zero from convolved time series with HRF. For each ROI and participant, the level of neural activity was averaged across voxels within each ROI using the PSC according to blocks of “crave” and “don’t crave” task conditions and the activity level was subtracted between “crave” and “don’t crave” conditions.

### 2.9 Offline analysis: Measures of functional connectivity

The level of functional connectivity was estimated using Pearson’s correlation coefficients between the average BOLD time series from pairs of multiple ROIs as shown in Figure 2C. The time points were defined based on volumes corresponding to the “crave” and “don’t crave” conditions and estimated coefficients were normalized using Fisher’s r-to-z transformation to ensure statistical comparability (Fox et al., 2009; Kim et al., 2015; Zar, 1996). Then, the functional connectivity level was averaged across voxels within each ROI for “crave” and “don’t crave” conditions and each participant, and the connectivity level was subtracted between “crave” and “don’t crave” conditions.

### 2.10 Offline analysis: Measures of activity-based classification

For individual activity-based classification as presented in Figure 2D, to compare “crave” and “don’t crave” conditions across the whole brain, PSC was normalized between 0 and 1 across volumes for each of the three runs. The scaling factors for the normalization in the training data were applied to the validation and test datasets. An explicit mask was applied to exclude the visual regions covering the bilateral calcarine, cuneus, lingual gyrus, and superior, middle, inferior occipital lobes from the Automated Anatomical Labeling (Tzourio-Mazoyer et al., 2002) and Brodmann area 17 (Brodmann, 1909). A ν-support vector machine (SVM) classifier with a linear kernel was used as implemented in the LIBSVM software toolbox (www.csie.ntu.edu.tw/~cjlin/libsvm). A nested *k*-fold (*k* = 10) nested cross-validation framework was adopted to optimize the latent parameter ν via a grid search using uniformly distributed candidate values between 0.1 and 0.8 with an interval of 0.1 (Chen et al., 2005). The cross-validation process was repeated 20 times using 20 randomly shuffled training, validation, and test sets to ensure reliability. For individual classification, the PSC were divided into 10 folds: 8 folds (n = 114 volumes) for training, 1 fold (n = 15) for validation, and 1 remaining fold (n = 15) for testing.

### 2.11 Offline analysis: Measures of connectivity-based classification

For individual connectivity-based classification to distinguish “crave” and “don’t crave” conditions at the whole-brain level as shown in Figure 2E, functional connectivity was calculated using seed-based analyses. To match the degrees of freedom between activity-based and connectivity-based classifications, temporally concatenated group independent component analysis (ICA) was applied using the group ICA of fMRI toolbox (GIFT; trendscenter.org/software/). A group ICA for each ROI was separately performed within each explicit ROI mask, and the number of independent components (IC; n = 144) for each ROI was determined with the same number of input samples (n = 144) used for activity-based classification. The ROIs including the ACC, PCC, left insula, right insula, precuneus, mOFC, and mSFG contained the number of voxels: 877, 276, 653, 590, 2145, 517, and 1622, respectively. Using the individual time series for each subject and run, the voxel-wise time series were concatenated across the volumes for “crave” and “don’t crave” conditions separately. These voxel-wise concatenated time series for each subject, run, and condition were entered as the input for group ICA. Therefore, group ICA across subjects and runs was independently performed for 14 total cases (i.e., 7 ROIs and 2 conditions). First, principal component analysis (PCA) was applied to reduce data dimension (# of PCs = 30 based on minimum description length (Rissanen, 1978)) for each subject and each run. Second, the dimension-reduced data at the subject level were concatenated across subjects and runs and an additional PCA was applied to reduce dimensions at the group level (# of PCs = 144). Next, ICA was used to estimate group-level ICs (# of ICs = 144) and back reconstruction based on the previous PCA steps was performed to extract subject-level ICs. All the ICs were used for subsequent analyses.

To estimate functional connectivity patterns using IC-wise time courses for each ROI, each of the IC time courses (n = 144) was extracted and an IC-wise connectivity from each ROI to the whole brain was estimated using Pearson’s correlation. The IC-wise coefficient was converted to *z*-score using Fisher’s *r*-to-*z* transformation (Fox et al., 2009; Kim & Lee, 2011; Zar, 1996) and then pseudo *z*-scoring was conducted across all the voxels of the whole brain such that the distribution of voxel values across the brain has a zero mean and unit variance (McKeown et al., 1998; Ng et al., 2012; Nichols & Holmes, 2002; Rier et al., 2023; Zunhammer et al., 2021). Then, the same framework (i.e., *k*-fold nested cross-validation) used in activity-based classification was used to examine the performance of the classifier using whole-brain functional connectivity patterns for each ROI. Subsequently, weight features from all the participants were normalized via pseudo *z*-scoring on the whole brain for each individual and were subjected to a one-sample *t*-test for group inference. One-way analysis of variance (ANOVA) was conducted with repeated runs as a within-subject independent variable to compare the weight features across the repeated runs.

To investigate which voxels highly contributed to classify the “crave” vs “don’t crave conditions from connectivity-based classification for each ROI, the segregated spatial maps of the ICs for each subject and run were used and those were multiplied by each corresponding support vector weight. The weighted spatial patterns were converted to pseudo z-scores within each ROI and then z-scored weighted patterns were averaged across permutations for each run and subject. One-way ANOVA was performed with repeated runs as a within-subject independent variable.

### 2.12 Offline analysis: Post hoc statistical tests

To evaluate statistical significance of “crave” vs “don’t crave” effects for each signal source, a post hoc paired *t*-test was conducted between pairs of the three runs. A linear regression model was performed to compare the performance of signal sources across the three runs. Bonferroni correction was applied to the *t*-test (n = 31) and the regression model based on the number of tests performed.

For comparison of “crave” vs “don’t crave” discriminability across signal sources, the average for each signal source was selected to conduct a two-way ANOVA with repeated runs as a within-subject independent variable and four signal sources as a within-subject independent variable. To do that, the neural activity differences based on PSC changes between conditions were averaged across the 7 ROIs, and the functional connectivity differences between conditions were averaged across the 21 pairs. For comparison of classification accuracy using whole-brain activity, test accuracy (rather than training accuracy) was used. For comparison of classification accuracies using functional connectivity patterns, test accuracies were averaged across the seven ROIs. In order to fairly compare across neural signal sources of different scales, classification accuracies were transformed into standardized effect sizes by subtracting chance accuracies (i.e., 50%) and dividing by standard deviation across subjects for each run and each classification. In addition, a paired *t*-test was applied to test which classification showed better discrimination between “crave” and “don’t crave” conditions. Additionally, a one-way ANOVA test was conducted using the first run only to compare “crave” vs “don’t crave” discriminability across signal sources as a within-subject factor, prior to any neurofeedback exposure.

## 3 Results

### 3.1 Overview

The primary question in this study is the comparison of “crave” vs “don’t crave” task conditions depending on the differing signal sources of neural activity, functional connectivity, activity-based classification, and connectivity-based classification. We also evaluated performance dependent on univariate or multivariate analytic approaches. For each signal source, therefore, post hoc *t*-tests between pairs of runs and linear regression were estimated to evaluate the statistical significance of “crave” vs “don’t crave” effects over multiple runs.

### 3.2 Neural activity changes

**Fig. 3.**
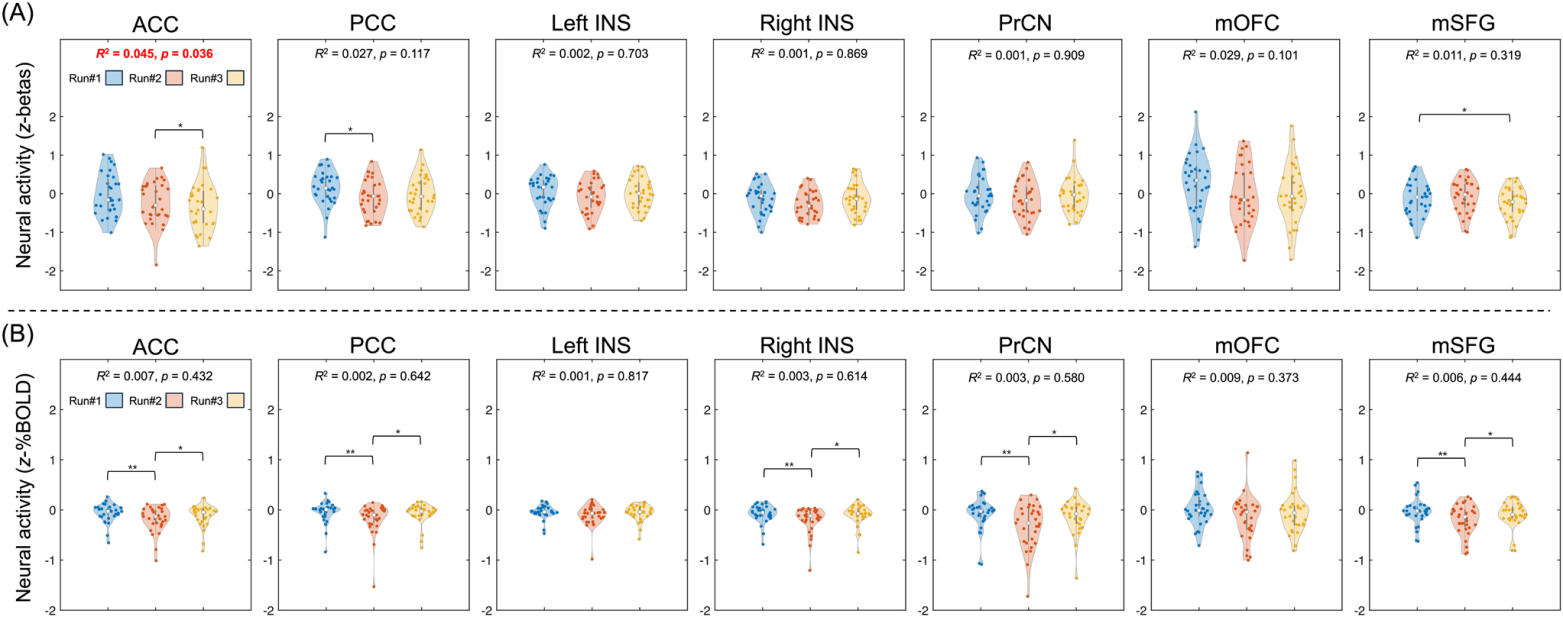
Neural activity based on (A) beta values from the contrast of “crave” and “don’t crave” conditions using general linear model and (B) difference of percentage BOLD signal changes between “crave” and “don’t crave” conditions. ACC, anterior cingulate cortex; PCC, posterior cingulate cortex; INS, insula; PrCN, precuneus; mOFC, medial orbitofrontal cortex; mSFG, medial superior frontal gyrus. *R*^2^ and corresponding *p*-value estimated using linear regression analysis and bold font indicating statistical significance. The asterisks show statistically significant difference using a paired *t*-test between pairs of the three runs. *p < 0.05; **p < 0.01.

Figure 3 shows the level of neural activity comparing three, repeated neurofeedback runs. When quantifying neural activity based on beta values from a contrast of “crave” and “don’t crave” conditions using the GLM, only the ACC among seven ROIs was identified with a significance (*R*^2^ = 0.045 and *p* = 0.036) of linear decrease across runs. Significant differences between pairs of runs were identified in ACC, PCC, and mSFG. When using the different PSC between “crave” and “don’t crave” conditions as neural activity, significant differences between pairs of runs were identified in ACC, PCC, right insula, PrCN, and mSFG.

### 3.3 Functional connectivity changes

**Fig. 4.**
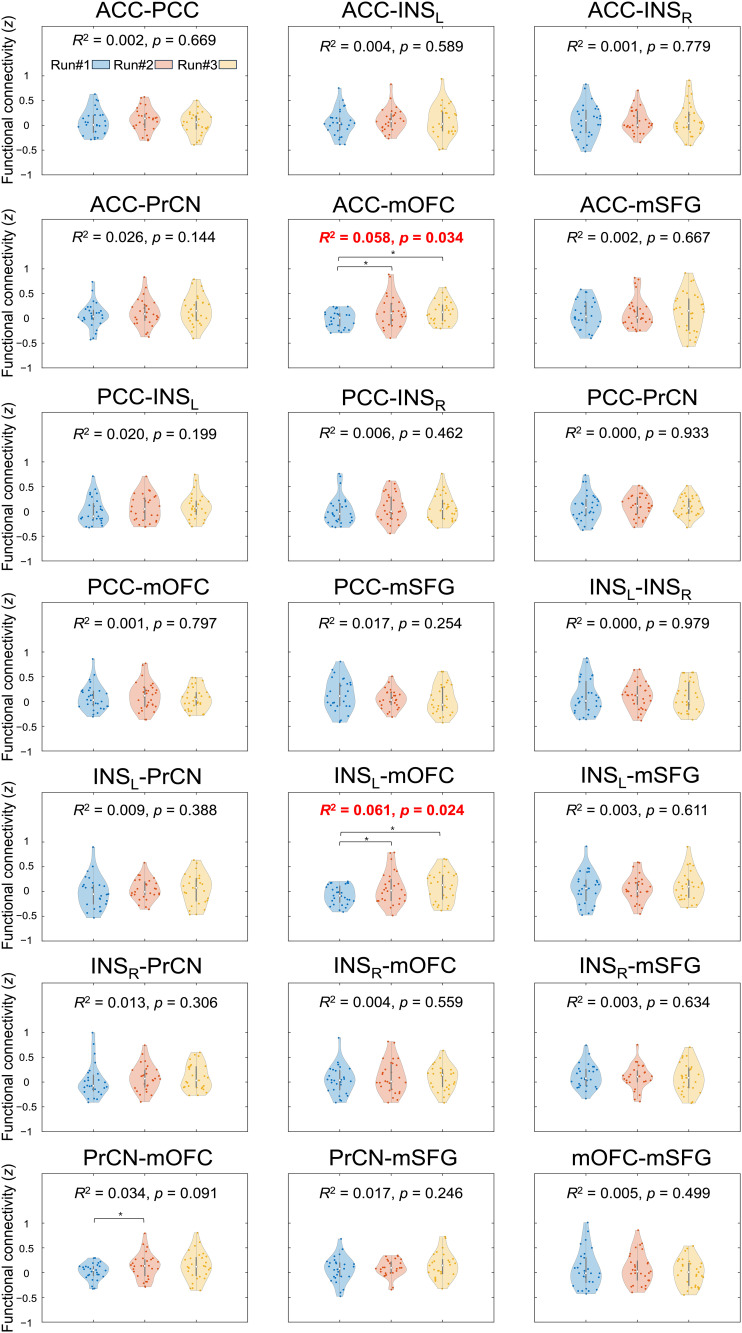
Functional connectivity difference between “crave” and “don’t crave” conditions. *R*^2^ and corresponding *p*-value estimated using linear regression analysis and red font indicating statistical significance. The asterisks show statistically significant difference using paired *t*-tests between pairs of the three runs. ACC, anterior cingulate cortex; PCC, posterior cingulate cortex; INS, insula; PrCN, precuneus; mOFC, medial orbitofrontal cortex; mSFG, medial superior frontal gyrus; L, left; R, right. *p < 0.05.

In Figure 4, the *z*-scored level of functional connectivity illustrates the estimated connectivity between pairs of the seven ROIs for each neurofeedback run. A statistically significant linear trend of connectivity difference between “crave” and “don’t crave” conditions across the repeated runs was found in the connectivity between pairs including ACC and mOFC (*R*^2^ = 0.058 and *p* = 0.034), as well as left insula and mOFC (*R*^2^ = 0.061 and *p* = 0.024). Significant differences in connectivity between pairs of runs were identified for ACC and mOFC, left insula and mOFC, and PrCN and mOFC.

### 3.4 Activity-based classification changes

**Fig. 5.**
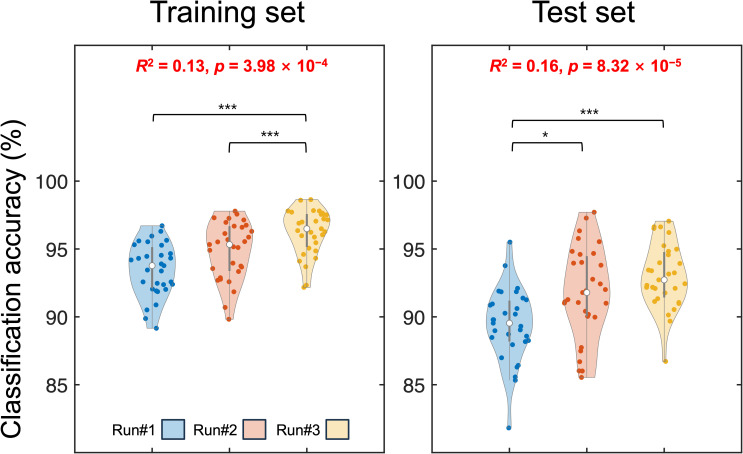
Classification accuracy of training and test set using whole-brain activity to classify “crave” and “don’t crave” conditions. *R*^2^ and corresponding *p*-value estimated using linear regression analysis and bold font indicating statistical significance. The asterisks show statistically significant difference using a paired *t*-test between pairs of the three runs. *p < 0.05; ***p < 0.001.

Figure 5 illustrates the accuracy from activity-based classification of whole-brain data. Statistical significance was found for the changes between “crave” and “don’t crave” conditions across the repeated runs for both training and test sets (*R*^2^ = 0.13, *p* = 3.98 × 10^−4^ and *R*^2^ = 0.16, *p* = 8.32 × 10^−5^, respectively). In the training set, the comparison of the first and third runs as well as the second and third runs was significant, while in the test set comparison of the first and second runs as well as the first and third runs was significant.

### 3.5 Connectivity-based classification changes

**Fig. 6.**
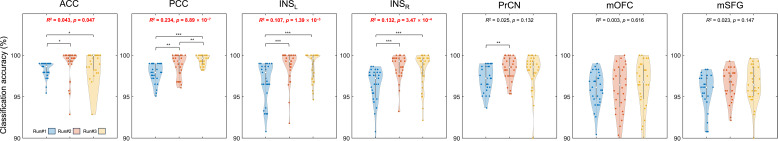
Classification accuracy of test set using functional connected patterns of each seed ROI to classify “crave” and “don’t crave” conditions. *R*^2^ and corresponding *p*-value estimated using linear regression analysis and bold font indicating statistical significance. The asterisks show statistically significant difference using a paired *t*-test between pairs of the three runs. ACC, anterior cingulate cortex; PCC, posterior cingulate cortex; INS, insula; PrCN, precuneus; mOFC, medial orbitofrontal cortex; mSFG, medial superior frontal gyrus; L, left; R, right. *p < 0.05; **p < 0.01; ***p < 0.001.

**Fig. 7.**
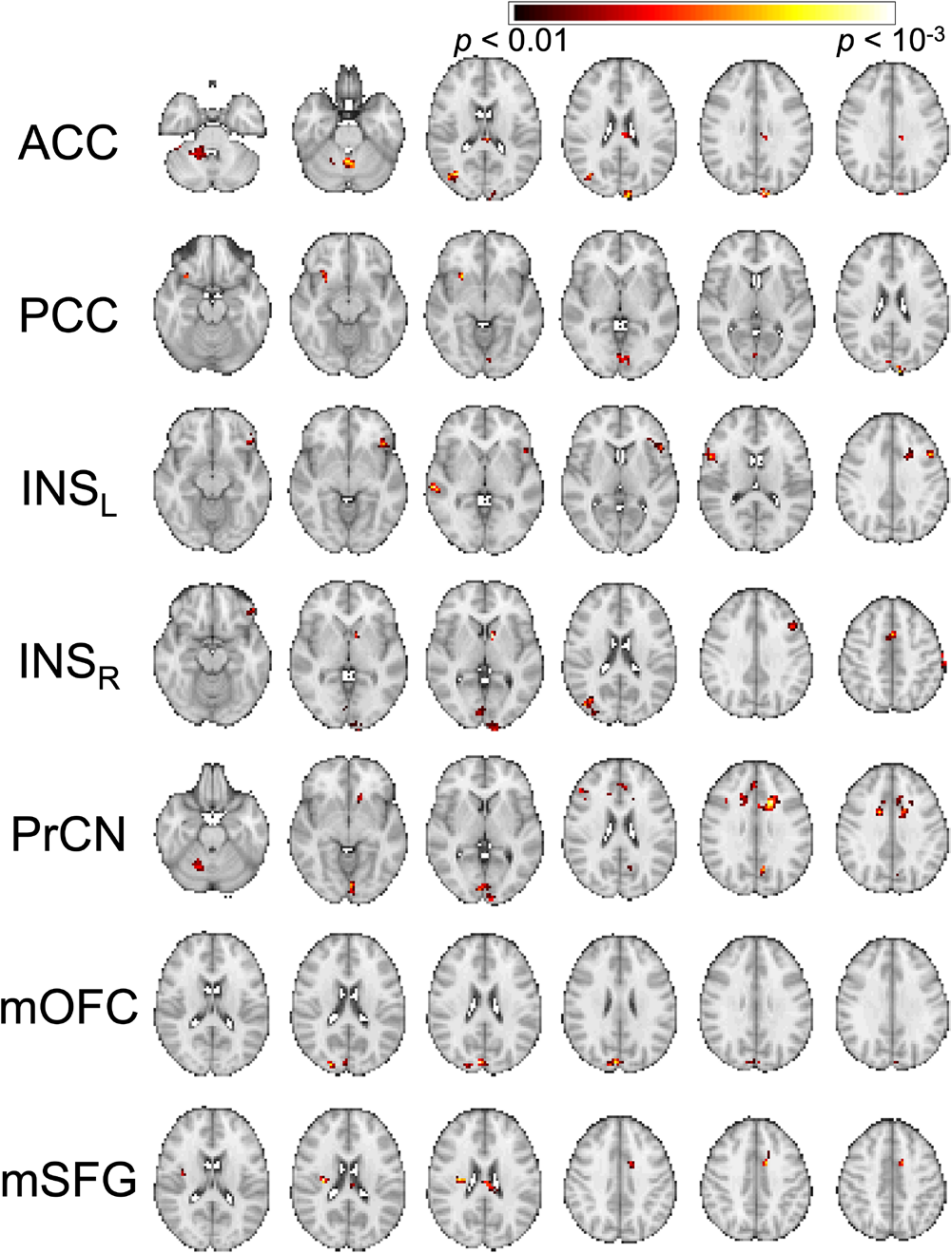
Results of one-way ANOVA test using weight patterns of connectivity-based classification across three repeated runs as a within-subject independent variable (details in Supplementary Material, Tables S1-S7). ACC, anterior cingulate cortex; PCC, posterior cingulate cortex; INS, insula; PrCN, precuneus; mOFC, medial orbitofrontal cortex; mSFG, medial superior frontal gyrus; L, left; R, right.

Figure 6 shows the accuracy of “crave” vs “don’t crave” connectivity-based classification for each ROI to the whole brain. A statistically significant linear increase across the repeated runs was found in the PCC (*R*^2^ = 0.234, *p* = 8.89 × 10^−7^), left insula (*R*^2^ = 0.107, *p* = 1.39 × 10^−3^), and right insula (*R*^2^ = 0.132, *p* = 3.47 × 10^−4^) and marginally significant in the ACC (*R*^2^ = 0.043, *p* = 0.047). The ACC, PCC, left/right insula, and PrCN were significantly reported from the comparison of pairs of the three runs. The spatial patterns via group inference using individual weight features are illustrated in Figure 7. From the ACC, connected brain regions were identified in the midbrain, subcortical areas, and parts of visual areas. The PCC showed connectivity with the right insula and parts of visual areas. The left insula was connected to the anterior insula, right middle temporal gyrus, bilateral middle frontal areas, and superior frontal gyrus. The right insula showed a connection to the lateral OFC, subcortical areas, parts of visual areas, lateral, and medial superior frontal gyrus. The PrCN was connected to the cerebellum, parts of visual areas, subcortical areas, lateral/medial middle/superior frontal gyrus. The mOFC showed the connection to visual areas. The mSFG was connected to the right temporal gyrus, subcortical areas, and middle cingulate cortex.

**Fig. 8.**
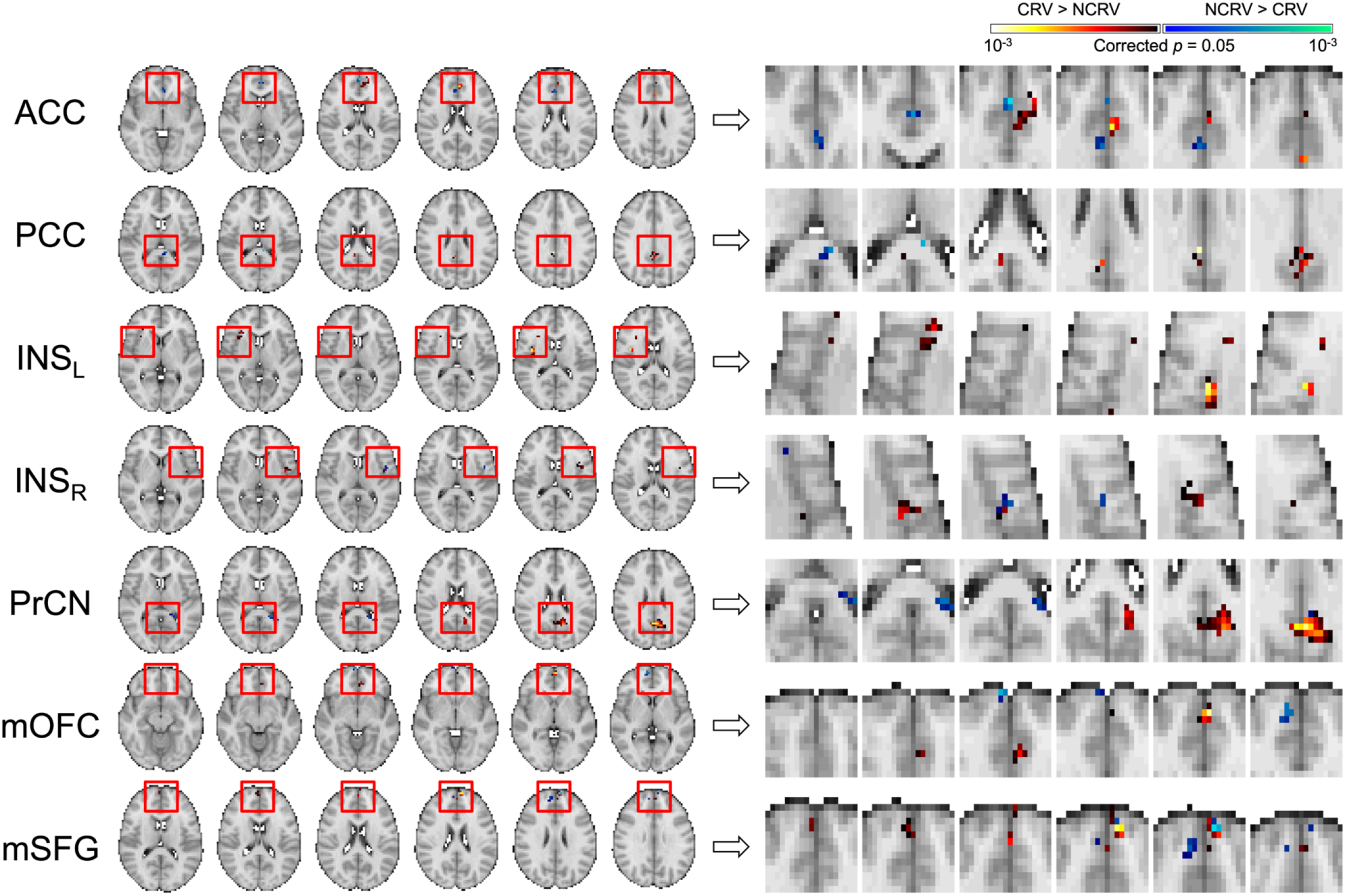
Contribution voxels within each ROI through one-way ANOVA test across repeated runs as a within-subject independent variable. Positive and negative values within statistically significant voxels indicating higher and lower weight values between “crave” and “don’t crave” conditions, respectively. ACC, anterior cingulate cortex; PCC, posterior cingulate cortex; INS, insula; PrCN, precuneus; mOFC, medial orbitofrontal cortex; mSFG, medial superior frontal gyrus; L, left; R, right.

The contributions of voxels within each ROI are represented in Figure 8. In the ACC and PCC, the superior and inferior parts were associated with “crave” and “don’t crave” conditions, respectively. Within the left insula, the anterior and posterior regions showed a relationship only in the “crave” condition. Within the right insula, the medial parts presented a mixture of “crave” and “don’t crave” conditions. In the PrCN, the posterior–superior parts showed the relationship with the “crave” condition but the anterior–inferior parts were related to the “don’t crave” condition. In the mOFC and mSFG, the inferior and superior parts showed a relationship with “crave” and “don’t crave” conditions, respectively.

### 3.6 Comparison across signal sources

Post hoc tests using ANOVA test across signal sources indicate significant main effects of repeated runs (*F* = 18.99, *p* = 1.45 × 10^-8^) and signal source (*F* = 665.87, *p* = 9.51 × 10^-99^). A significant interaction effect between signal sources and repeated runs was also observed (*F* = 8.99, *p* = 3.68 × 10^-9^). Parsing of this interaction for each signal source revealed significant main effects of run derived from multivariate sources (*F* = 3.35, *p* = 0.02 for activity-based classification and *F* = 21.13, *p* = 4.41 × 10^-12^ for connectivity-based classification) but not from univariate sources. Connectivity-based classification showed significantly higher accuracy than activity-based classification (*t* = 16.39, 11.00, and 14.92, *p* = 1.63 × 10^-16^, 4.71 × 10^-12^, and 2.02 × 10^-15^ for the first, second, and third runs, respectively). One-way ANOVA test using the first run revealed a significant main effect of signal sources (*F* = 138.86, *p* = 9.13 × 10^-22^).

## 4 Discussion

### 4.1 Summary

Our prior work investigated a machine learning-based rtfMRI-NF method to regulate “crave” and “don’t crave” brain patterns across repeated neurofeedback training (Kim et al., 2024). Here we re-analyzed those data and evaluated performance for discriminating “crave” and “don’t crave” conditions depending on the neural signal source. Multivariate machine learning-based measurements showed superior performance compared with neural activity or functional connectivity, where better performance was quantified as greater statistical significance for distinguishing “crave” from “don’t crave” conditions between pairs of repeated runs or across repeated runs. Of note, the machine learning model with seed-based whole-brain connectivity patterns showed superior performance than other signal sources; variability of performance was evident across different seed brain regions.

### 4.2 Signal source-dependent neurofeedback performance

Many rtfMRI-NF studies have adopted various signal sources to reflect neurofeedback information such as neural activity (Canterberry et al., 2013; Hartwell et al., 2016; Karch et al., 2019; Young et al., 2017; Zich et al., 2020; Zotev et al., 2024), functional connectivity (Kim et al., 2015; Megumi et al., 2015; Papoutsi et al., 2020; Taebi et al., 2024; Tsuchiyagaito et al., 2021; Zich et al., 2020), effective connectivity (Koush et al., 2013; Morgenroth et al., 2020), and machine learning models (Collin et al., 2022; Fede et al., 2023; LaConte, 2011; Lorenzetti et al., 2018; McDonald et al., 2017; Shibata et al., 2011; Weaver et al., 2020). Neurofeedback derived from brain connectivity or machine learning measures from multiple brain areas may have an advantage over activity-based neurofeedback, as post hoc results of neurofeedback training indicate changes in connectivity between bilateral brain regions or brain features at the whole-brain level. However, few studies have investigated the comparison of performance across signal sources (Fede et al., 2023; Haugg et al., 2020; Kim et al., 2015; Lorenzetti et al., 2018; Papoutsi et al., 2020). For example, Fede and colleagues report a negative association between SVM measures and alcohol craving scores; but measures from activity-based neurofeedback show a strong correlation with craving scores (Fede et al., 2023). This discrepancy may result from the techniques used to define multiple brain regions for activity-based neurofeedback or over-performed training for SVM-based neurofeedback. In this study, we compared four different signal sources (neural activity, functional connectivity, activity-based SVM, and connectivity-based SVM) using offline analyses of our prior rtfMRI neurofeedback data in smokers regulating “crave” vs “don’t crave” brain responses. Our findings suggest that machine learning approaches outperform “crave” vs “don’t crave” discriminability based on within-ROI activity and between-ROIs connectivity. In addition, connectivity-based multivariate pattern analysis can provide model-free features that capture heterogeneity in group-level functional connectivity, in line with prior findings (Nieto-Castanon, 2022). The superior performance of connectivity-based features may reflect the engagement of large-scale brain networks involved in craving regulation, such as the cognitive control, salience, and default mode networks. These networks coordinate distributed neural processes underlying the monitoring and regulation of craving-related affective states. Connectivity-based measures may, therefore, provide a more comprehensive representation of the dynamic interactions among these systems, capturing complex, distributed neural strategies beyond what single-region activity measures could establish. This interpretation is consistent with prior evidence that effective craving regulation relies on coordinated activity between prefrontal control regions and subcortical or insular areas associated with interoceptive and emotional processing (Chung et al., 2023; Hartwell et al., 2016; Karch et al., 2019; Pandria et al., 2023). Given this, the present data suggest potential utility of using multiple brain information sources in neurofeedback training. However, further studies are required to determine whether differing neural signal sources, or combination of sources, lead to improved real-time neuromodulation and/or whether potential improved neuromodulation yields changes in subjective craving or confers therapeutic benefits.

### 4.3 Variability of functional networks within and/or across brain regions

We defined seven brain regions previously associated with smoking craving: anterior cingulate cortex, posterior cingulate cortex, left and right insular cortex, precuneus, medial orbitofrontal cortex, and medial superior frontal gyrus. As expected, the performance of discriminating “crave” vs “don’t crave” conditions through repeated neurofeedback runs varied depending on the ROI; nonetheless, the connectivity-based MVPA model from the posterior cingulate cortex and bilateral insula presented superior significance over other ROIs and other signal sources. These findings align with previous studies indicating that key brain regions, such as the insula and posterior cingulate cortex, may have crucial roles in controlling smoking cravings (Ghahremani et al., 2021; Janes et al., 2015; Kim et al., 2020; Moran‐Santa Maria et al., 2015; Naqvi et al., 2007) or mediating functional processes in positive and negative affect (Brewer & Garrison, 2014; Garavan, 2010; Naqvi & Bechara, 2009). For example, Naqvi and Bechara proposed a model indicating the insular cortex as a target for addiction therapies, in which a historical perspective has an evidence in integrating interoceptive states into conscious urges and decision-making processes (Naqvi & Bechara, 2009). Meanwhile, the posterior cingulate cortex has been related to associative learning in the context of affective reactivity (Brewer & Garrison, 2014). Given the earlier findings of smoking craving-related studies, the key brain regions are reasonable seed areas to examine functional networks of the whole brain; however, we note that definition of relevant seed regions likely also depends on the cognitive process or clinical population of interest.

In addition, we identified the contributions of within-ROI voxels on the whole-brain functional connectivity patterns to classify “crave” vs “don’t crave” conditions. Of note, the dorsal regions of ACC (Fedota & Stein, 2015; Hayashi et al., 2013; O’Neill et al., 2023), PCC (Engelmann et al., 2012; Fedota & Stein, 2015), and PrCN (Claus & Weywadt, 2020; Engelmann et al., 2012; Fedota & Stein, 2015) were identified to be significantly associated with smoking craving along with the ventral parts of mOFC (Fedota & Stein, 2015; Hayashi et al., 2013) and mSFG (Engelmann et al., 2012; Fedota & Stein, 2015; Hayashi et al., 2013). Compared with “crave”, “don’t crave” task condition significantly identified the ventral parts of ACC, PCC, and PrCN and the dorsal parts of mOFC and mSFG. This can be considered as evidence for a potential pathway to be reversely connected between the dorsal and ventral parts of anterior and posterior smoking-related brain regions depending on smoking craving and not-craving. Furthermore, the left insula showed a dominant relationship with smoking craving, but the right insula presented mixed patterns between smoking “crave” and “don’t crave” task conditions. Taken together, the observed contributions within each ROI might indicate potential relevance for defining seed voxels for estimation of functional connectivity patterns to classify conditions.

### 4.4 Potential limitations and future studies

Studies implementing real-time fMRI neurofeedback have adopted different signal sources for providing feedback information, and the present data suggest multivariate analyses may have particular strength for differentiating processes related to craving. However, it is important to note that the signals investigated here were originally acquired in the context of a neurofeedback task wherein the feedback was generated based on a machine learning model trained and updated based on whole-brain activity-based classification (Kim et al., 2024). Despite this potential bias, for the present secondary analysis, the best discrimination between “crave” and “don’t crave” conditions across repeated neurofeedback runs was found from the functional connectivity patterns-based classification. In addition to present the changes of neural signal itself, the behavioral and demographic information might be used to provide evidence of actual relationship with the neurofeedback effect across repeated training. Future work may assign experimental groups to prospectively receive feedback from independent different signal sources such as activity, connectivity, or machine learning-based measures solely. We note that the age range of participants in the present study was relatively broad. To evaluate potential confounding effects, we examined relationships between each neural signal source and age, gender, and cigarette use measures (detailed in Supplementary Material, S.1.1, S.2.1, and Fig. S1). While no significant relationships were observed for the majority of correlation analyses between neural signal source and these variables, connectivity-based accuracy and age were significantly related for the second and third runs. Therefore, further investigation is warranted to examine associations of functional connectivity patterns and age in a larger study sample (Geerligs et al., 2017; Heckner et al., 2021; Millar et al., 2022), with larger variability of age across smoking behavior measures.

We emphasize that the models in this study relied on data generated from “crave” and “don’t crave” instructional labels used in our previously published real-time fMRI study (Kim et al., 2024). Additional regression analyses between each neural signal source and post hoc questionnaires identified a significant and specific relationship between functional connectivity pattern-based classification accuracy and smoking craving during the neurofeedback runs (details in Supplementary Material, S.1.2, S.2.2, and Fig. S2). In addition, unsupervised classification of “crave” and “don’t crave” conditions separately showed excellent performance (mean accuracy > 85%; details in Supplementary Material, S.1.3, S.2.3, and Fig. S3). Together, these results support the consistency of “crave” and “don’t crave” brain states and a potential functional relationship between successful modulation of craving-related brain patterns and changes in subjective craving. Future studies may directly test this possibility with real-time assessment of subjective craving as task instructional labels change.

In addition, the block-based design in our experimental paradigm, which involved counter-balanced smoking-related images and fixation images, may be potentially weaker for identifying connectivity components than continuous or longer-lasting stimuli. A previous pilot study compared the degree of regulation from tinnitus patients between continuous and intermittent neurofeedback, and its performance with continuous neurofeedback showed improvement across multiple sessions over intermittent neurofeedback (Emmert et al., 2017). More generally, these data together provide evidence that the selection of signal source for neurofeedback should be considered in the context of differing experimental designs as well as stimulus types. We do note that while it is promising that the functional connectivity pattern-based classification was associated with subjective craving ratings, improved discriminability of “crave” and “don’t crave” neural responses may not be related to variation in online neural modulation or modulation of subjective craving. Future studies employing causal perturbation methods or longitudinal neurofeedback training are needed to determine whether voluntary regulation of these neural targets translates into changes in subjective responses or behavior.

## 5 Conclusion

We have presented evidence supporting the feasibility of discriminating “crave” vs “don’t crave” task conditions in smokers, comparing performance across activity, connectivity, activity-based multivariate measures, and connectivity-based multivariate neural measures. The findings highlight not only the potential advantage of machine learning-based real-time fMRI neurofeedback for modulating brain features but also suggest that connectivity-based multivariate sources could provide particular benefit for intervening on cognitive processes through neurofeedback.

## Supplementary Material

Supplementary Material

## Data Availability

Data underlying this article are made accessible through the Virginia Tech Data Repository at https://doi.org/10.7294/32374128.
