# Supplementary material for: Comparison of neural signal sources for discriminating “crave” and “don’t crave” task conditions: Implications for fMRI neurofeedback
Source: Imaging Neurosci (Camb). 2026 Jun 18;4:IMAG.a.1277. doi: 10.1162/IMAG.a.1277 (PMC13281776; doi:10.1162/IMAG.a.1277)
Supplement: Supplementary Material [file IMAG.a.1277_supp.pdf]

Supplementary Materials

S.1 Methods

S.1.1 Evaluation of potential confounding effects

To evaluate potential confounding effects of age, sex, and smoking-related information, correlation analyses were conducted between each of the five neural signal sources and age, sex, number of cigarettes used yesterday, and number of cigarettes smoked per day. Pearson's correlations were calculated between each signal source and age, number of cigarettes used yesterday, and number of cigarettes smoked per day, respectively. The relationship between each signal source and sex as a dichotomous variable was estimated using point biserial correlation (Kornbrot, 2014; Tate, 1954).

S.1.2 Regression analysis using behavioral measurements and neural signal sources

After MRI scanning, participants were asked to answer a series of questions about their subjective experiences during the imaging portion of the study. The items included Likert-style ratings (range 1 – 5; 1, not at all; 2, a little; 3, moderately; 4, quite a bit; 5, extremely) of: 'how much did you experience cigarette craving during' the (i) 'crave' condition with no slider, (ii) 'don't crave' condition with no slider, (iii) 'crave' condition with slider, and (iv) 'don't crave' condition with slider. A regression analysis for each run was conducted to estimate the relationship between each neural signal source (neural activity, functional connectivity, activity-based classification accuracy, and connectivity-based classification accuracy) and subjective craving rating during the neurofeedback runs. The Pearson's correlation coefficient and corresponding *p*-value are reported for each run.

S.1.3 Unsupervised one-class classification

"The consistency of classes identified in the supervised classification between neural 'crave' and 'don't crave' conditions was further evaluated by implementing unsupervised one-class classification of the 'crave' and 'don't crave' conditions separately. The same input as for binary classification was used, such that the percent signal change was normalized between 0 and 1 across volumes (i.e., number of volumes = 144 for each of the 'crave' and 'don't crave' conditions of each of the three runs) in the training data. The normalization of volumes for each condition was performed with a range from 0 to 1 across the number of voxels × the number of volumes used, and the scaling factors for this normalization were applied to the validation and test data. A ν-SVM classifier with a linear kernel was used, and the latent parameter ν was optimized via a grid search using uniformly distributed candidate values (from 0.1 to 0.8, with an interval of 0.1) (Chen et al., 2005). Then, 10-fold nested cross-validation (same as used in binary classification) examined the consistency across all the activity patterns for each 'crave' and 'don't crave' condition.

S.2 Results

S.2.1 Relationship between neural signal sources and demographic variables

Figure S1 shows the relationship between each neural signal source and demographic and cigarette use variables. Two significant relationships were observed in which connectivity-based classification accuracies were correlated with age for the second and third runs. No other significance was found.

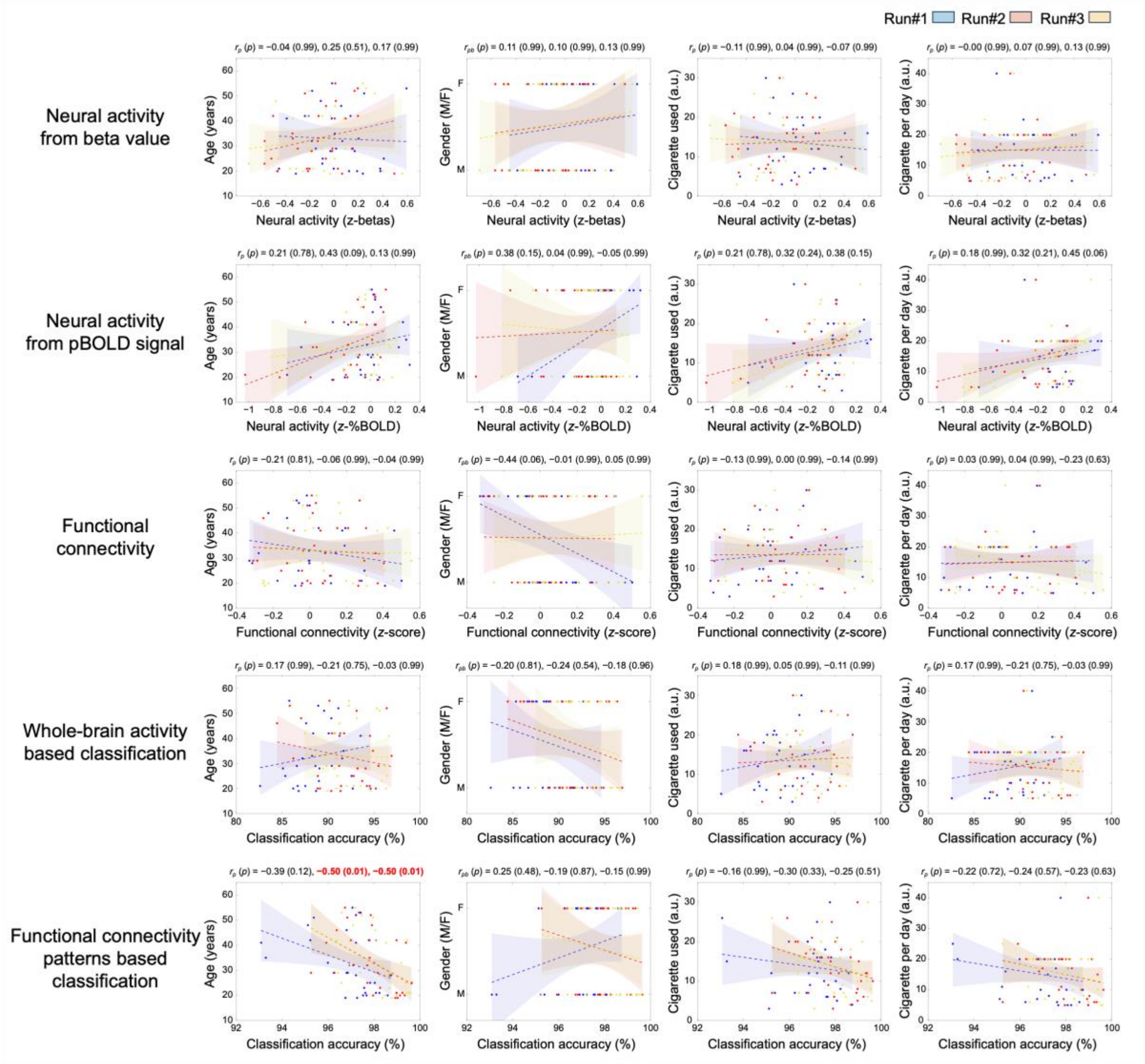

Figure S1. Relationship between each neural signal source and age, sex, number of cigarettes used yesterday, and number of cigarettes smoked per day. Regression lines and the 95% confidence interval are presented for each run. The Pearson's correlation coefficients and corresponding *p*-values for each neural signal source and age, number of cigarettes used yesterday, and number of cigarettes smoked per day are shown for each run at the top of each subplot, and for each neural signal source and sex, the point biserial correlation and corresponding *p*-values are shown. The *p*-values from the correlation analyses were Bonferroni-corrected by multiplying by the number of runs. %BOLD, percentage blood-oxygen-level-dependent signal; r_p, Pearson's correlation coefficient; r_{pb}, point biserial correlation.

S.2.2 Relationship between subjective measures and neural signal sources

The distributions of subjective craving ratings, assessed after scanning and querying about the neurofeedback runs for the 'crave' and 'don't crave' conditions, are shown in Figure S2(A). Participants reported significantly less craving during the 'don't crave' neurofeedback condition compared with during 'crave' neurofeedback (p = 0.005 from a paired t-test). Figure S2(B) shows the relationship between functional connectivity based accuracy and subjective smoking craving. The average of connectivity-based classification of 'crave' vs 'don't crave' conditions during the second and third runs (neurofeedback runs) was significantly correlated with participants' smoking craving difference scores (r = 0.41, p = 0.02).

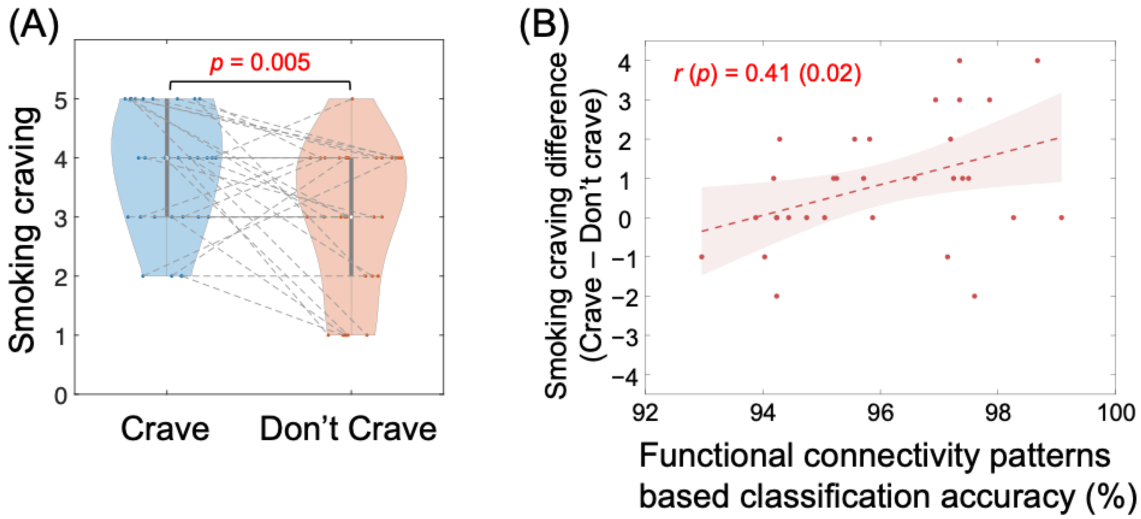

Figure S2. (A) Subjective craving rating when queried about craving during the 'crave' and 'don't crave' neurofeedback blocks, respectively (B) relationship between participants' subjective smoking craving difference scores ('crave' – 'don't crave' condition craving ratings) and neural functional connectivity patterns based classification accuracy of 'crave' and 'don't crave' conditions. The regression line and the 95% confidence interval are presented. r, Pearson's correlation coefficient.

S.2.3 Unsupervised classification of 'crave' and 'don't crave' conditions

Figure S3 shows the results of one-class classification analyses to evaluate the consistency of activity patterns within the 'crave' and 'don't crave' conditions, respectively. Overall classification accuracies over 85% were observed (89.48 ± 4.32, 90.06 ± 3.02, and 87.92 ± 4.18 for the first, second, and third runs in the 'crave' condition, respectively and 89.02 ± 3.63, 89.35 ± 4.33, and 88.27 ± 4.27 for the first, second, and third runs in the 'don't crave' condition, respectively).

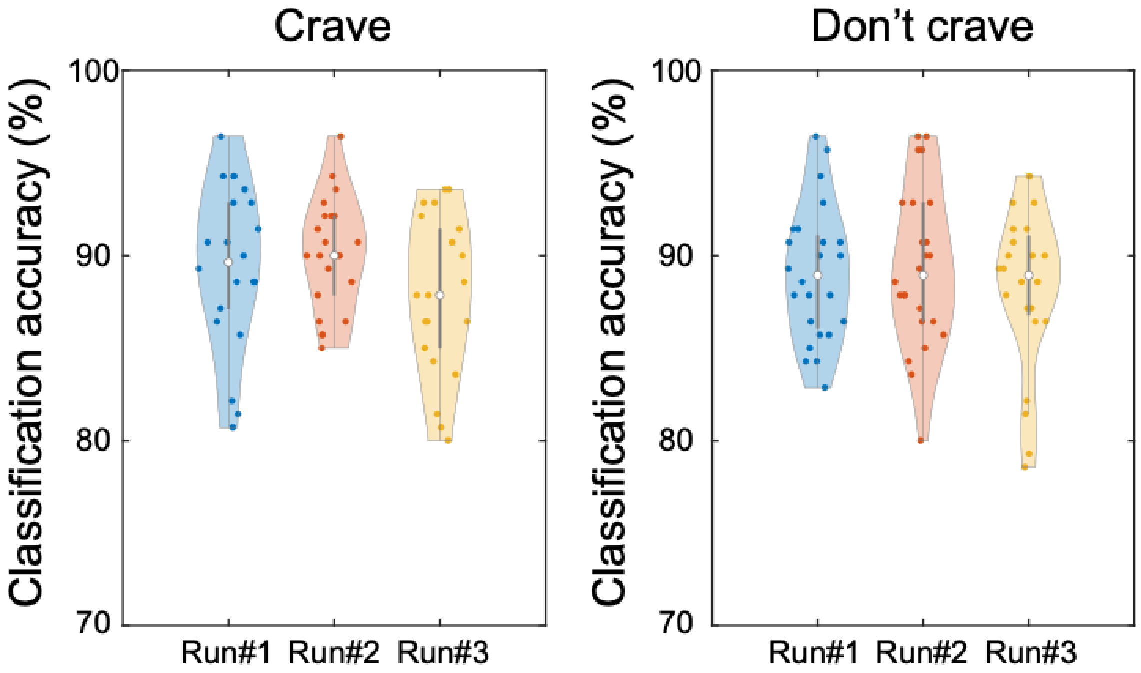

Figure S3. Unsupervised classification accuracy of test sets using whole-brain activity to test 'crave' and 'don't crave' conditions for each run.

Table S1. Brain regions identified from ANOVA test for the anterior cingulate cortex as seed region (visualized at the first row in Figure 7). Resulting *p*-values were corrected using 10,000 random permutations and voxel-clusters with a minimum of 20 contiguous voxels survived from corrected $p < 0.05$ were reported.

Cluster index	*Brain region*	*Side*	*MNI coordinate* **(x, y, z in mm)**	*Number of voxels*	*Peak F-score*	*Corrected p-value*
1	Cerebellum Crus2	R	42, −40, −37	23	4.64	0.020
2	Cerebellum 6	R	21, −46, −34	37	3.43	0.047
3	Vermis 8	L/R	0, −55, −28	36	4.82	0.017
4	Cuneus	L	−9, −94, 29	68	5.31	8.39×10^{-4}
5	Pulvinar medial thalamic nuclei	L/R	−3, −28, 17	42	4.00	0.035
6	Middle occipital gyrus	R	33, −70, 17	38	5.19	0.020

L, left; R, right; MNI, Montreal Neurological Institute

Table S2. Brain regions identified from ANOVA test for the posterior cingulate cortex as seed region (visualized at the second row in Figure 7). Resulting *p*-values were corrected using 10,000 random permutations and voxel-clusters with a minimum of 20 contiguous voxels survived from corrected $p < 0.05$ were reported.

Cluster index	*Brain region*	*Side*	*MNI coordinate* **(x, y, z in mm)**	*Number of voxels*	*Peak F-score*	*Corrected p-value*
1	Insula	R	27, 17, −10	33	5.12	0.011
2	Calcarine	L/R	3, −76, 11	79	3.70	0.043
3	Cuneus	L/R	−9, −97, 23	26	4.46	0.023

L, left; R, right; MNI, Montreal Neurological Institute

Table S3. Brain regions identified from ANOVA test for the left insula as seed region (visualized at the third row in Figure 7). Resulting *p*-values were corrected using 10,000 random permutations and voxel-clusters with a minimum of 20 contiguous voxels survived from corrected $p < 0.05$ were reported.

Cluster index	*Brain region*	*Side*	*MNI coordinate* **(x, y, z in mm)**	*Number of voxels*	*Peak F-score*	*Corrected p-value*
1	Inferior orbitofrontal cortex	L	−42, 26, −10	42	4.86	0.016
2	Superior temporal gyrus	R	60, −25, −1	21	6.11	3.18×10^{-4}
3	Triangular part of inferior frontal gyrus	L	−48, 23, 5	33	4.16	0.030
4	Opercular part of inferior frontal gyrus	R	54, 11, 14	33	4.92	0.014
5	Superior frontal gyrus	L	−21, 20, 32	53	4.05	0.034
6	Middle frontal gyrus	L	−45, 14, 35	31	5.10	0.011

L, left; R, right; MNI, Montreal Neurological Institute

Table S4. Brain regions identified from ANOVA test for the right insula as seed region (visualized at the fourth row in Figure 7). Resulting *p*-values were corrected using 10,000 random permutations and voxel-clusters with a minimum of 20 contiguous voxels survived from corrected $p < 0.05$ were reported.

Cluster index	*Brain region*	*Side*	*MNI coordinate* **(x, y, z in mm)**	*Number of voxels*	*Peak F-score*	*Corrected p-value*
1	Lateral orbitofrontal cortex	L	−48, 38, −16	21	4.55	0.022
2	Middle occipital gyrus	L	−12, −106, 2	36	4.60	0.021
3	Calcarine	R	6, −88, 5	24	4.07	0.033
4	Caudate	L	−9, 5, 2	20	7.73	5.62×10^{-6}
5	Middle occipital gyrus	R	42, −76, 20	67	5.56	4.81×10^{-4}
6	Middle frontal gyrus	L	−42, 17, 38	27	4.68	0.019
7	Supplementary motor area	L/R	0, 8, 47	30	6.17	2.66×10^{-4}
8	Inferior parietal lobule	R	−60, −19, 50	29	4.97	0.013

L, left; R, right; MNI, Montreal Neurological Institute

Table S5. Brain regions identified from ANOVA test for the precuneus as seed region (visualized at the fifth row in Figure 7). Resulting *p*-values were corrected using 10,000 random permutations and voxel-clusters with a minimum of 20 contiguous voxels survived from corrected $p < 0.05$ were reported.

Cluster index	*Brain region*	*Side*	*MNI coordinate* **(x, y, z in mm)**	*Number of voxels*	*Peak F-score*	*Corrected p-value*
1	Cerebellum Crus2	L	42, −43, −40	21	4.16	0.030
2	Cerebellum 6	R	18, −55, −28	27	5.19	0.001
3	Fusiform gyrus	L	33, −64, −16	28	5.27	9.48×10^{-4}
4	Caudate	R	−15, 23, −7	22	3.54	0.045
5	Calcarine	L/R	−3, −82, −7	78	4.94	0.014
6	Precuneus	L	−9, −64, 29	26	4.88	0.015
7	Triangular part of inferior frontal gyrus	R	39, 23, 26	31	3.97	0.036
8	Middle cingulate cortex	L	−18, 17, 29	98	6.31	9.36×10^{-5}
9	Anterior cingulate cortex	R	15, 5, 35	46	5.38	7.87×10^{-4}
10	Anterior cingulate cortex	L/R	0, 38, 26	22	4.06	0.034
11	Inferior parietal lobule	L	−57, −43, 50	32	5.19	0.001

L, left; R, right; MNI, Montreal Neurological Institute

Table S6. Brain regions identified from ANOVA test for the medial orbitofrontal cortex as seed region (visualized at the sixth row in Figure 7). Resulting *p*-values were corrected using 10,000 random permutations and voxel-clusters with a minimum of 20 contiguous voxels survived from corrected $p < 0.05$ were reported.

Cluster index	*Brain region*	*Side*	*MNI coordinate* **(x, y, z in mm)**	*Number of voxels*	*Peak F-score*	*Corrected p-value*
1	Cuneus	L/R	6, −85, 23	48	5.57	4.49×10^{-4}

L, left; R, right; MNI, Montreal Neurological Institute

Table S7. Brain regions identified from ANOVA test for the medial superior frontal gyrus as seed region (visualized at the seventh row in Figure 7). Resulting *p*-values were corrected using 10,000 random permutations and voxel-clusters with a minimum of 20 contiguous voxels survived from corrected $p < 0.05$ were reported.

Cluster index	*Brain region*	*Side*	*MNI coordinate* **(x, y, z in mm)**	*Number of voxels*	*Peak F-score*	*Corrected p-value*
1	Insula	R	30, −16, 20	28	6.05	3.56×10^{-4}
2	White matter	L/R	−3, −22, 23	26	4.12	0.031
3	Middle cingulate cortex	L	−12, 5, 41	25	5.04	0.012

L, left; R, right; MNI, Montreal Neurological Institute

References

Chen, P. H., Lin, C. J., & Schölkopf, B. (2005). A tutorial on ν-support vector machines. *Applied Stochastic Models in Business and Industry*, *21*(2), 111-136. https://doi.org/10.1002/asmb.537

Kornbrot, D. (2014). Point biserial correlation. *Wiley StatsRef: Statistics Reference Online*. https://doi.org/10.1002/9781118445112.stat06227

Tate, R. F. (1954). Correlation between a discrete and a continuous variable. Point-biserial correlation. *The Annals of mathematical statistics*, *25*(3), 603-607. https://doi.org/www.jstor.org/stable/2236844
